# Transcriptome analysis of the honey bee fungal pathogen, *Ascosphaera apis:* implications for host pathogenesis

**DOI:** 10.1186/1471-2164-13-285

**Published:** 2012-06-29

**Authors:** R Scott Cornman, Anna K Bennett, K Daniel Murray, Jay D Evans, Christine G Elsik, Kate Aronstein

**Affiliations:** 1 Honey Bee Research Unit, USDA-ARS, Weslaco, TX, 78596, USA; 2 Honey Bee Research Laboratory, USDA-ARS, Beltsville, MD, 20705, USA; 3 Department of Biology, Georgetown University, Washington, DC, 20057, USA

## Abstract

**Background:**

We present a comprehensive transcriptome analysis of the fungus *Ascosphaera apis*, an economically important pathogen of the Western honey bee *(Apis mellifera)* that causes chalkbrood disease. Our goals were to further annotate the *A. apis* reference genome and to identify genes that are candidates for being differentially expressed during host infection versus axenic culture.

**Results:**

We compared *A. apis* transcriptome sequence from mycelia grown on liquid or solid media with that dissected from host-infected tissue. 454 pyrosequencing provided 252 Mb of filtered sequence reads from both culture types that were assembled into 10,087 contigs. Transcript contigs, protein sequences from multiple fungal species, and *ab initio* gene predictions were included as evidence sources in the Maker gene prediction pipeline, resulting in 6,992 consensus gene models. A phylogeny based on 12 of these protein-coding loci further supported the taxonomic placement of *Ascosphaera* as sister to the core Onygenales. Several common protein domains were less abundant in *A. apis* compared with related ascomycete genomes, particularly cytochrome p450 and protein kinase domains. A novel gene family was identified that has expanded in some ascomycete lineages, but not others. We manually annotated genes with homologs in other fungal genomes that have known relevance to fungal virulence and life history. Functional categories of interest included genes involved in mating-type specification, intracellular signal transduction, and stress response. Computational and manual annotations have been made publicly available on the Bee Pests and Pathogens website.

**Conclusions:**

This comprehensive transcriptome analysis substantially enhances our understanding of the *A. apis* genome and its expression during infection of honey bee larvae. It also provides resources for future molecular studies of chalkbrood disease and ultimately improved disease management.

## Background

Chalkbrood disease of the honey bee is caused by *Ascosphaera apis* (Maassen ex Claussen), [1] a filamentous fungus that exclusively infects bee larvae[2]. Adult honey bees are not susceptible to infection but can disperse spores, particularly via food sharing [3]. After ingestion, fungal spores germinate in the larval gut, mycelia cross the gut lining and proliferate through the body cavity, invading all internal organs until the larva dies of systemic mycosis. The fungus then continues development and nutrient acquisition inside the cadaver. Mycelia eventually emerge from the body cavity and form masses of ascomata on the outer surface. If environmental conditions allow, cadavers desiccate to form chalkbrood ‘mummies’ that can contain millions of ascospores [4]. In accordance with this life history, *A. apis* spores germinate best in nearly-anaerobic conditions, while mycelial growth and sporulation is aerobic and can be maintained in axenic culture [2].

Chalkbrood can lead to heavy losses in affected honey bee colonies, but disease severity depends on multiple factors including: age of larvae, dose of inoculums, temperature, and humidity. Chalkbrood outbreaks have most frequently been recorded in cool and humid climates, although they may also occur in hot and dry climates [5]. Currently, management of chalkbrood (reviewed by [5]) focuses on sanitation methods that target the long-lived spores, improved management of symptomatic disease, and use of disease-resistant honey bees. A wide spectrum of fungicides has been tested against *A. apis*, but none of them are approved for use in beehives [6]. Natural compounds with anti-fungal properties are also being investigated. Therefore, there is a continued need for cost-effective and widely applicable treatments that do not leave chemical residues in bee products. A better understanding of the mechanisms of pathogenesis would help advance this goal.

Genome-scale sequence analysis is a powerful and efficient strategy to identify genes involved in the complex interactions between host and pathogen. In this study, we have performed high-throughput sequencing to compare the *A. apis* transcriptome as it is expressed during axenic culture versus controlled infection of larvae. This strategy can help identify genes that may be responsive to the host environment or are otherwise important for pathogenicity, although subsequent replication is needed to validate these candidates. Our data are used to support revised gene models, to evaluate the completeness of the current genome assembly [7], and to quantify differential read counts between growth conditions. We also identify components of key molecular pathways such as signal transduction and mating type. Our results extend previous genomic analyses of *A. apis*[7,8], providing additional resources for investigating pathogenesis and, ultimately, identifying effective treatment strategies.

## Results and discussions

### Transcriptome assembly

A total of 992,370 454 reads were obtained for the two barcoded samples, with a mean length of 341.7 bp. The combined assembly produced 21,744 transcript contigs grouped into 9,984 isogroups (clusters of contigs that partially overlap, but which cannot be further collapsed under the assembly parameters) as well as 103 ungrouped contigs. Of these 10,087 transcript contigs, 9,534 (94.5%) had a Mega BLAST match to the reference genome assembly (E-value < 1.0e-10). Of the remaining 553 transcript contigs, 315 (57%) had a BLASTX match to a RefSeq fungal protein (E < 1.0e-5). Few transcript contigs were unalignable due to low-complexity sequence: only ten consisted of more than 25% low-complexity sequence as classified by the mdust package (http://compbio.dfci.harvard.edu/tgi/software/) under default parameters. Thus, most unaligned transcript contigs appear to be from valid *A. apis* genes that are not represented in the current genome assembly. These transcripts were included in the read-mapping analysis below but not in the gene-prediction pipeline.

### Gene prediction

Using Maker [9] to integrate *ab initio* predictions, transcript alignments, and protein homologs, we obtained 6,992 gene models, which is within the range of other sequenced ascomycetes [10,11]. A GBrowse [12] viewer of these models and other genome features are publicly available on the Bee Pests and Pathogens section of the Hymenoptera Genome Database [13] (http://hymenopteragenome.org/beebase/?q=bee_pathogens). We found some evidence of alternative splicing from mapped transcript contigs and expect that deeper sequence coverage or transcript-specific analyses would confirm its occurrence given results in other fungi (e.g. [14]). However, the small number of Maker-predicted alternative splice sites did not appear to be robust as a group (for example, concatenation of exons from distinct genes was a more plausible explanation than alternative splicing in some cases), such that we have conservatively removed alternative transcripts from this analysis.

To further evaluate the completeness of the *A. apis* genome assembly, we used the Core Eukaryotic Genes Mapping Approach (CEGMA) tool [15], with the CEGMA subset of 248 widely conserved eukaryotic core genes that are considered to have low frequencies of gene family expansion http://(http://korflab.ucdavis.edu/Datasets/genome_completeness/). We estimate that 203 of 248 CEGMA genes are complete in our gene list, and an additional ten genes are represented as fragments, for a total representation of 86% of the eukaryotic core gene set. A larger set of 441 core genes is also available from the CEGMA website for *Neurospoa crassa*, for which we identified 379 probable orthologs (86%) in *A. apis*. Thus, both eukaryotic and fungal core gene sets are equally represented in our annotations. These results are concordant with the fraction of non-matching transcript contigs, and suggest that less than 15% of the true gene content remains unassembled.

### Phylogeny

**Figure 1:**
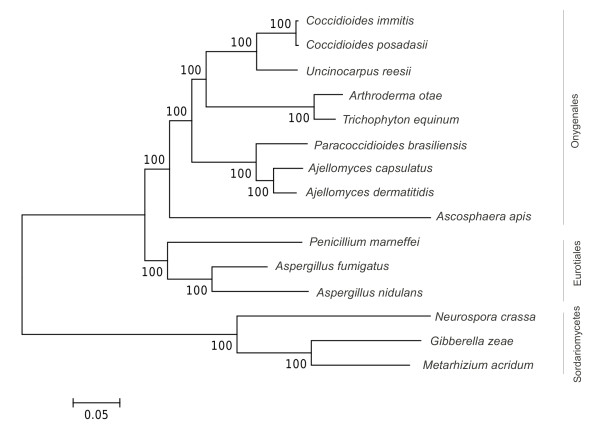
**Phylogeny of A. apis and 14 other Eurotiomycete fungi.** Twelve conserved protein-coding loci were used to clarify the phylogenetic position of A. apis. Amino-acid sequences were aligned with MUSCLE and the best fitting evolutionary model (JTT + G + I) was selected with MEGA5. The unrooted maximum likelihood tree is based on this model with five rate categories; bootstrap support from 1,000 replicates is shown.

Geiser and colleagues [16] used ribosomal sequence to estimate the phylogeny of the Eurotiomycetes, the ascomycete class to which *Ascosphaera* has been assigned. Their results indicated a position within order Onygenales, although the authors noted that support for the placement of *Ascosphaera* was not strong. To further clarify the evolutionary position of *A. apis* within the Eurotiomycetes, we used 14 representative species with sequenced genomes to create a phylogeny from the concatenated alignment of 12 conserved proteins (see Methods). The resulting maximum-likelihood tree (Figure 1) placed *A. apis* in a position congruent with that estimated by [16], i.e. within the Onygenales and not the sister clade Eurotiales. However, *A. apis* is the earliest branching of the Onygenales species included in this phylogeny. Furthermore, while each of the 12 loci produced tree topologies for the other 14 species that were concordant with the concatenated data set, the position of *A. apis* within the Eurotiomycetidae was variable for each individual locus (results not shown). This phylogenetic instability appears to be due to a higher level of sequence divergence along the *A. apis* lineage, as indicated by the long branch leading to *A. apis* in Figure 1.

### Domain analysis

We used HMMER [17,18] to identify Pfam domains in our gene models with an expectation threshold of E-value< 0.1 and compared the distribution to other sequenced and annotated ascomycetes. Given the smaller gene number in *A. apis*, it is not surprising that this species had fewer domain types (Table 1). Of greater interest are changes in domain number that suggest gene-family expansion and contraction within a phylogenetic lineage. To investigate this, we identified Pfam domains present in *A. apis* for which there were more than five additional genes with that domain in all other ascomycete genomes examined (Table 1). The *A. apis* lineage appears to have experienced reductions in several common domains, most strikingly the cytochrome p450 and protein kinase domains. Others that appear reduced include domains characteristic of transcription factors (zinc finger and fungal-specific transcription factor) and several categories of the Rossmann fold superfamily of nucleotide-binding domains (e.g., methyltransferase and FAD/NAD-binding domains). In contrast, no Pfam domain was identified for which there were more than five additional copies in *A. apis* compared with the other six genomes. Thus, there have not been major expansions of gene families with described domains since the divergence of the *A. apis* lineage from these other species.

**Table 1:** **Pfam domains less abundant in*Ascosphaera apis*compared with related ascomycete species**

**Pfam domain**	***A. apis***	***A. capsulatus***	***A. otae***	***A. fumigatus***	***C. immitis***	***M. acridum***	***N. crassa***
p450	11	29	82	77	40	87	42
Pkinase	54	146	176	113	138	147	109
Ankyrin	25	40	60	61	46	84	46
F-box	15	31	43	30	36	34	33
Fungal transcription factor	32	41	60	142	53	108	60
All zinc finger	210	324	292	439	329	331	304
All FAD binding	26	54	60	113	54	88	71
All NAD binding	20	28	50	82	36	66	41
All methyltransferase	37	52	93	94	59	88	79
All Rossman fold	234	283	379	609	338	520	406
Total domain types	2,627	2,720	2,917	3,103	2,833	3,029	3,038
Total domain matches	7,467	8,484	9,708	12,205	9,062	11,027	9,856
Undetected domains found in all other species	187	126	17	31	85	40	26
Total proteins searched	6,992	9,313	8,765	9,631	10,440	9,850	9,841

*Domains were selected in which* A. apis *has at least six fewer proteins with a match (E < 0.1) than all other species examined. Only domains present in all seven ascomycete species were considered (*Ascosphaera apis, Ajellomyces capsulatus, Arthroderma otae, Aspergillus fumigatus, Coccidioides immitis, Metarhizium acridum, Neurospora crassa*).*

**Figure 2:**
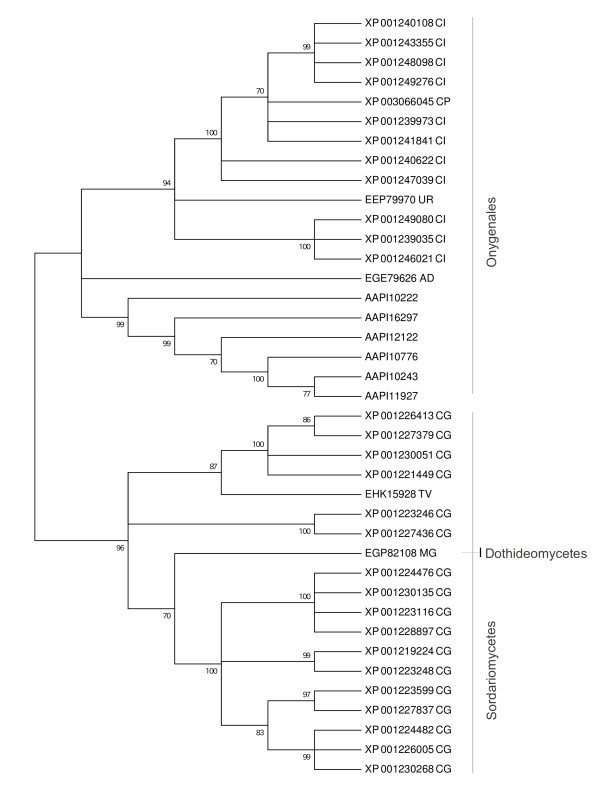
**Phylogeny of a novel gene family present in A. apis and other Ascomycete fungi.** An unrooted, neighbor-joining phylogeny of A. apis gene models and BLASTP-matching GenBank accessions. The tree is based on the most conserved region, illustrated in Figure 3, although other regions of sequence similarity occur. Only the topology of the tree is shown, with all nodes collapsed that had less than 70% bootstrap support. Two-letter codes after each accession signify the species of origin: AD, Ajellomyces dermatitidis; CH, Chaemtomium globosum; CI, Coccidioides immitis; CP, Coccidioides posadasii; MG, Mycosphaerella graminicola, TV, Trichoderma virens; UR, Uncinocarpus reesii.

**Figure 3:**
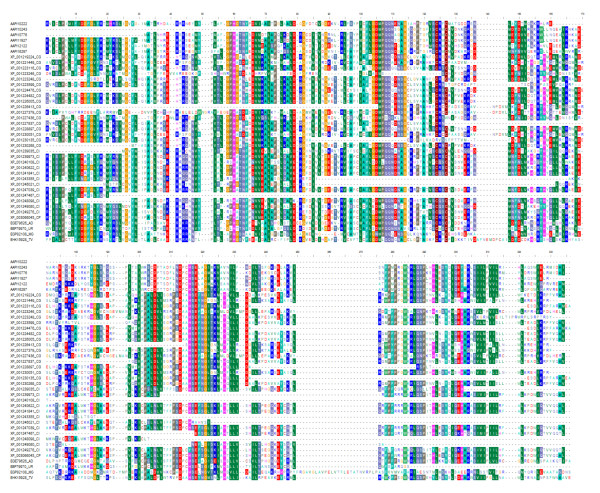
**Core conserved region characterizing a novel gene family identified in A. apis that also occurs in other ascomycete fungi.** Representative A. apis predicted proteins and GenBank accessions were aligned with MUSCLE and then manually trimmed to the most conserved region. Two-letter codes after each accession signify the species of origin: AD, Ajellomyces dermatitidis; CH, Chaemtomium globosum; CI, Coccidioides immitis; CP, Coccidioides posadasii; MG, Mycosphaerella graminicola, TV, Trichoderma virens; UR, Uncinocarpus reesii.

We searched for novel gene families within *A. apis* by using BLASTClust to group all proteins without a significant Pfam domain. One family of homologous sequences was identified that included seven *A. apis* genes predictions. However, two genes, AAPI10243 and AAPI10242, may be N- and C-terminal fragments of a single protein, and AAPI10243 also shared a long stretch of identical nucleotide sequence with AAPI11927, suggesting a possible assembly error. Thus, the family may contain only five distinct members. BLASTP against the NCBI nr database identified homologs of this gene family in other ascomycete genomes, but with an unusual distribution. Single homologs were found in *Neurospora crassa, Ajellomyces dermatitidis, Uncinocarpus reesii*, and the ascomycete *Trichoderma virens*, and multiple matches were found in *Coccidioides posadasii* and the ascomycete *Mycosphaerella graminicola*, some of which appeared to be fragments as annotated. In contrast, the gene family is greatly expanded in *Chaetomium globosum* and *Coccidioides immitis*, with 18 and 19 matching protein predictions, respectively. Most of these homologs are shown in a neighbor-joining phylogeny in Figure 2 (some partially aligning predictions were excluded due to potential annotation errors). This phylogeny is based on an alignment of BLASTP-matching peptides in GenBank, which was then trimmed to include only the most conserved region, illustrated in Figure 3. Distances were based on the JTT matrix and nodes with less than 70% support from 1,000 replicates were collapsed in the consensus phylogeny. The clustering of paralogs within species and the overall concordance of the gene and species phylogenies (Figures 1 and 2, see also the phylogeny of [16]) suggests independent expansions of the gene family within *Ascosphaera, Chaetomium*, and *Coccidioides*. Indels were common in the conserved region shown in Figure 3, suggesting that these genes may be rapidly evolving or not under strong purifying selection. There was no indication that these proteins are secreted or have transmembrane domains, however, based on SignalP [19] and TMHMM [20] domain searches.

A TBLASTN search using this region identified highly significant matches in other ascomycete species as well, several of which were apparent pseudogenes based on the presence of nonsense mutations. One TBLASTN match was in the subtelomeric region of *N. crassa* chromosome IVL, suggesting that members of this gene family may occur in subtelomeric repeats. If so, this may explain the idiosyncratic pattern of expansions observed, as subtelomeric regions frequently harbor gene amplifications and are often correlated with fungal niche adaptation [21]. Interestingly, no cDNA reads mapped to this gene family. However, four of the homologs in *C. immitis* were among the 51 genes found to be under positive selection in that species by [22].

### Pathway annotation and analysis

#### Virulence factors

Filamentous fungi produce a variety of secondary metabolites, many of which are involved in virulence [23,24] . The ability of an entomopathogenic fungus to infect its insect host is thought to depend on the coordinated activity of these virulence molecules together with mechanical pressure of hyphae on the exoskeleton and/or gut peritrophic membrane. In support of this model, we detected several transcripts involved in the production of *A. apis* hydrolytic enzymes, listed in Additional file 1: Table S1. For example, we identified genes encoding chitinases, proteases and esterases, degrading enzymes that could be involved in both host invasion and escape processes. We have also identified several genes encoding homologs of a cutinase transcription factor, Ctf1, indicating the ability of this fungus to utilize different nutrient substrates, including plant cutin, a variety of lipids as well as synthetic triacylglycerols and esters [25].

Furthermore, our updated genome annotation reveals a number of genes encoding homologs of well-known toxins and a diverse family of enzymes, some of them involved in the Aflotoxin (AF)-Sterigmatocystin (ST) biosynthesis pathway (*AflRStcUNor-1SteW, OmtA, OrdA*), HC-toxin biosynthesis *(Tox A, ToxG, ToxD, ToxF* and *Hts1),* and a super killer protein 3 (*Ski3*) (Additional file 1: Table S1). In addition to genes involved in toxin biosynthesis, our gene set includes a large variety of genes involved in transcription, translation and biosynthesis of enzymes that have also been implicated in virulence. The list includes an extracellular glucoamylase, 3 chitinases, 16 amidases, 30 esterases, 42 proteases, 24 lipases and others (Additional file 1: Table S1). A large number of these enzymes were found to be expressed in the infected host, which may implicate their role in host pathogenesis. Among them, a member of the metallopeptidase family M28 containing a transferrin receptor-like dimerisation domain (IPR007365) and a protease-associated PA domain (IPR003137) similar to a metallopeptidase found in *C. immitis* as well as vacuolar endopeptidase Pep2, a broad specificity secreted hydrolase that has been implicated in *Aspergillus fumigatus* virulence [24]. Another interesting example is that of the phospholipases (*plcA, plcB, plcC* and *plcD)* previously identified in *Mycobacterium tuberculosis*[26]. The *A. apis* genome encodes multiple genes homologous to those found in *M. tuberculosis, Neosartorya ficheri* and *Phytophtora infestans* (Additional file 1: Table S1).

Many virulence factors produced by *A. apis* have been previously identified using enzymatic activity assays and electrophoresis. The list includes galactosidases, glucosidases, catalases, phoshphatases, DNAses, and RNAses [27,28]. Among those, 12 enzymes and 285 isozymes were found in *A. apis* and closely related fungi, *Ascosphaera aggregata* and *Ascosphaera proliperda*[27,29]. Enzymatic activity of 11 enzymes (i.e. protease, *β-N-*acetylglucosaminidase, alkaline phosphatase, esterase, esterase lipase, leucine arylamidase, valine arylamidase, acid phosphatase, naphthol-AS-BI-phosphohydrolase*, β-*glucosidase and *α-*mannosidase) has been characterized by [28]. However, it is very difficult to compare our findings to those previously reported since earlier investigations mostly focused on the enzymatic activity, and therefore lacked gene and/or protein related data [27,28]. Furthermore, some of the previously published data is not supported by our findings. For example, the lack of chitinase activity in *A. apis* has been determined experimentally by many investigators, that led to a conclusion that *A. apis* does not digest insect chitin [27,30-32]. In the absence of chitinase activity, the N-acetyl-β-glucosaminidase in conjunction with hyphal pressure has been suspected in aiming penetration of the peritrophic matrices lining the gut epithelium [27]. However, our gene predictions identified at least three different glycosyl hydrolases with the GH18 Pfam domain characteristic of class III and class V chitinases (http://www.cazy.org) (Additional file 1: Table S1). GH family 18 chitinases are frequently implicated in the mycoparasitic activity by the degradation of exogenous chitin [33] and thus their presence in *A. apis* revives the possibility that these enzymes are involved in host pathogenesis. We detected four and two sequence reads from this gene in axenic culture and infection, respectively, indicating that it is not exclusively expressed during infection. However, additional work is needed to definitively determine whether any of these chitinases are involved in penetrating the peritrophic matrix of the honey bee larva. It is noteworthy that GH18 chitinases are highly sensitive to allosamidin (β-D-allopyranoside), a potent chitinase inhibitor (http://www.reference.md) and therefore may be a potential target for fungal control.

### Signal transduction

Fungi have sophisticated and conserved signalling cascades to sense and respond to a rapidly changing environment by regulating essential functions such as melanin formation, mating, virulence, and morphogenesis. Signal transduction pathways described in fungi include the Mitogen-Activated Protein Kinase (MAPK) pathway, cAMP-dependent protein kinase pathway (PKA), and calcineurin-Ca^2+^-calmodulin-activated phosphatase signalling. Among those, the MAPK cascade is a key pathway involved in stress responses and fungal pathogenicity in a wide range of pathogenic fungi. Here we identified several components of this pathway, including several MAP kinase homologs (*Hog1, MapK, Spm1*), MAP kinase kinase (*Mkk1*) and stress activated MAP kinase kinase kinase (*Win1*) (Additional file 1: Table S1).

### RNA interference pathway

Post-transcriptional gene silencing is a part of a broad host defence response against nucleic acid invaders in most eukaryotic organisms, including filamentous fungi [34-36]. Although eukaryotic genomes vary in the number of genes involved in the RNAi machinery, generally, filamentous ascomycete fungi encode two homologs each of the core RNAi proteins Dicer (Dcl) and Argonaute (Ago). However, some fungi, such as *Aspergillus nidulans*, appear to encode only a single copy of the Dcl and Ago proteins.

Our genome analysis showed that *A. apis* contains most of the core RNAi components, including two Dicer genes (*Dcl-1* and *Dcl-2*) and one Argonaute (*Ago1*), but not *Ago2,* similar to *A. nidulans* (Additional file 1: Table S1). The sequence for this gene is present in neither the genome assembly nor in the non-aligned transcript contig sequences. It is possible that some components of the RNAi pathway might have been lost in *A. apis* during its divergence; with other family members joining the RNA-induced silencing (RISC) complex to assume the function of the missing family members [35]. Presence of the core RNAi components in the *A. apis* genome and RdRP homolog can be exploited for development of an RNAi-based control strategy of this bee pathogen [36].

### Sexual reproduction

Sexual reproduction in ascomycetous fungi is governed by a single mating type (MAT) locus with two idiomorphs. Although flanking regions are frequently conserved, idiomorphic alleles can differ greatly in structure and gene content, but minimally encode a transcription factor with either an α-box domain or a high-mobility-group (HMG) domain [37-40]. These transcription factors activate alternative pathways for mating-type development.

The *A. apis* MAT-2 allele, which contains the HMG-domain gene, was previously identified [8]. However, those authors were unable to identify the MAT-1 allele either through sequence analysis of the *A. apis* genome assembly or degenerate PCR, suggesting that the allele was not adequately covered by genome sequencing or was too divergent to be annotated or amplified. In this sequencing effort we have identified a transcript contig that is a BLASTX match to an *A. capsulatus* α-box protein (GenBank accession ABO87596), but does not align to the genome assembly. To determine whether this transcript is part of the *A. apis* MAT-1 allele, we designed primers to amplify this sequence from genomic DNA, under the assumption that it is flanked by the genes *Sla2* and *Apn2* as in the MAT-2 idiomorph [8].

**Figure 4:**
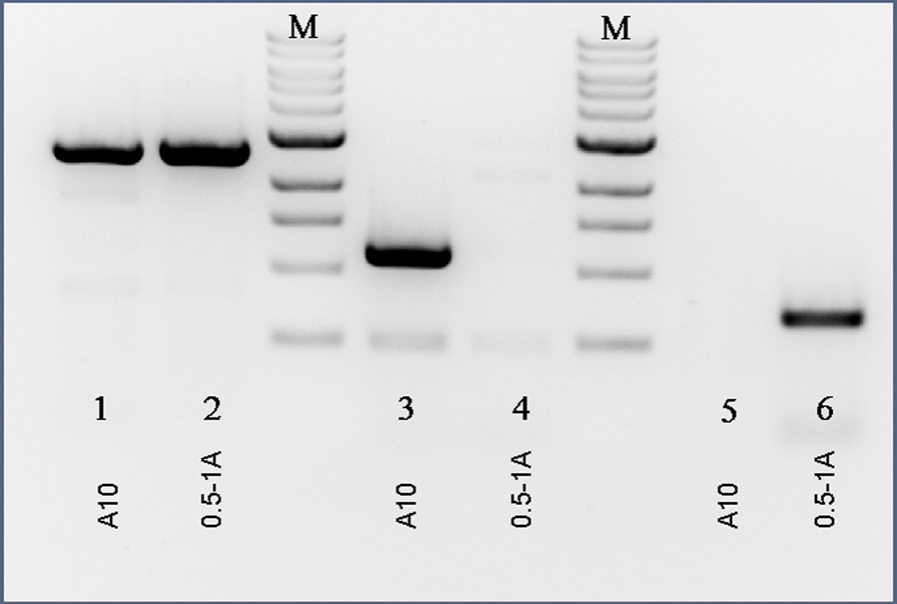
**Amplification of alternative mating type alleles from A. apis genomic DNA.** Lanes 1–2: amplification of a 2.8 kb span of the MAT locus from a MAT-1 strain (labeled “A10”) and a MAT-2 strain (labeled “0.5-1A”), respectively, using the primer combination 5′ flaking MAT2 (Scaf74) and DNALR1. Lanes 3–4: primers targeting the α-box-encoding transcript identified from transcriptome sequencing (see text) amplified a product from the MAT-1 but not the MAT-2 strain. Lanes 5–6: primers targeting the HMG-domain-encoding gene of the MAT-2 allele amplified a product from the MAT-2 but not the MAT-1 strain.

**Figure 5:**
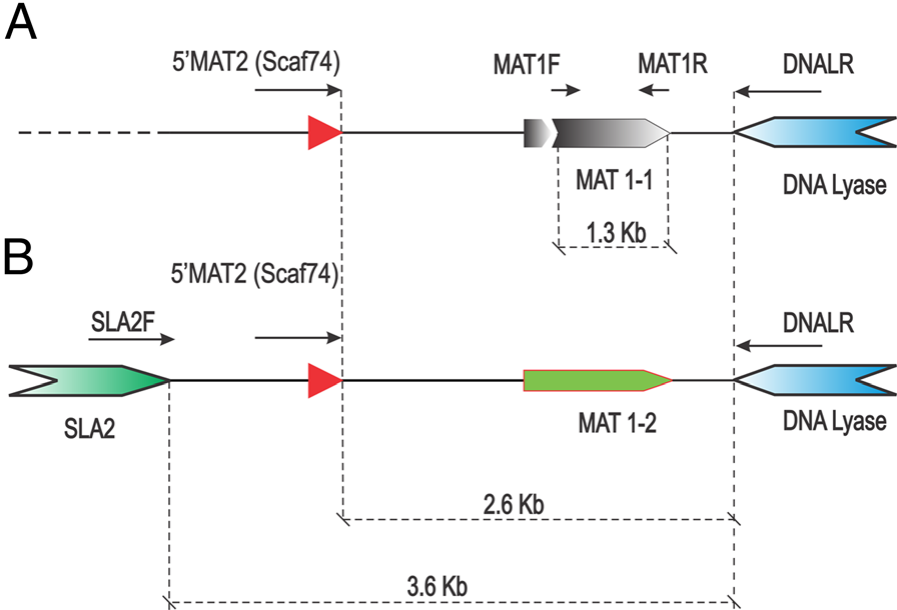
**Inferred structure of the A. apis mating type locus.** MAT-1 and MAT-2 mating types are represented by isolates “A10” and “0.5–1A”, respectively. A) MAT-1 allele containing an α-box domain transcription factor. B) MAT-2 locus containing a HMG domain transcription factor. Arrows indicate the location and direction of PCR primers used in this study. GenBank or the Bee Pests and Pathogens (AAPI) accession numbers are provided in Additional file 1:Table S1.

Figure 4 summarizes our PCR analysis of the MAT locus structure for MAT-1 and MAT-2 strains of *A. apis*. A forward primer annealing upstream of the MAT-2 HMG-domain gene (5′MAT2) and a reverse primer annealing to the *Apn2* gene (primer DNALR1) [8] produced a 2.8 kb product in both strains (lanes 1 and 2 of Figure 4) as expected. However, no PCR product could be amplified from the MAT-1 strain using the *Sla2*/*Apn2* primer combination, suggesting that the *Sla2* gene is not within the idiomorph-specific region and is divergent in sequence, position, or orientation. We were able to amplify products specific to the putative *Mat1-1* α-box transcript contig and the *Mat1-2* HMG-domain gene only from the respective strains, confirming that the α-box transcript derives from the MAT-1 allele of the MAT locus. A diagram of the inferred structure is presented in Figure 5.

### Comparative gene expression

The present study was primarily designed to improve the annotation of *A. apis*. The data can in principle be used to estimate differences in transcript abundance between axenic culture and host infection, thereby identifying candidate host-responsive genes for further study. However, without additional replication, it is impossible to calculate a variance for individual transcripts and thus distinguish between environmental and stochastic effects. An implementation of Fisher’s Exact Test can be used to estimate the significance of differential read count abundance [41], but for unreplicated data sets we expect the resulting P-values to be strongly influenced by the high stochasticity typical of genome-scale expression patterns. This leads to a somewhat arbitrary, *post hoc* choice of P-value threshold for identifying differential expression. Given these considerations, we adopted an *ad hoc* strategy to identify genes that are strong candidates for having host-responsive expression, but nonetheless requiring further validation. In addition to calculating the P-value of Fisher’s Exact Test (with multiple-test correction) with the edgeR package [41], we calculate a variance in log_2_ (ΔRPKM) as a function of transcript length using a sliding-window approach (see Materials and Methods). Genes that were both two standard deviations from the local mean and significant by Fisher’s Exact Test were considered to be candidate differentially expressed genes. We limited our sample to genes with at least 50 mapped reads in at least one growth condition (1,928 gene models and unmapped transcript contigs, or 25.5% of the total).

**Figure 6:**
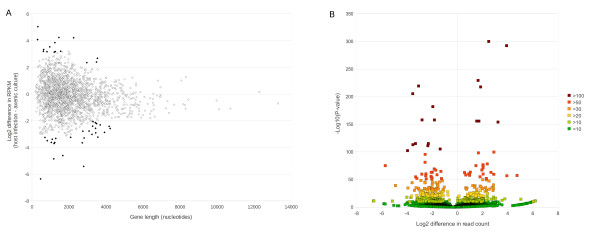
**Differential gene expression.** Differential counts of reads mapping to A. apis gene models between culture types, with positive values indicating higher abundance in host infection relative to axenic culture. A) Differential counts of reads, normalized by both total reads mapped and transcript length (RPKM), between host infection and axenic culture is plotted on a log_2_ scale on the vertical axis. Values are plotted as a function of gene length in nucleotides because the variance in RPKM is greater for shorter genes. Black points are greater than two standard deviations from the mean RPKM for a sliding window of 500 genes, ranked by length. B) Differential counts of reads normalized for total mapped reads in each library but not for length of predicted transcript. Log_2_ differential is on the horizontal axis and significance by Fisher’s Exact Test with multiple test correction is on the vertical axis. Colors indicate progressive ranges of probability of the null hypothesis (equal abundance in both samples), plotted as the -log10 (P-value) for clarity.

Figure 6 shows two views of the distribution of differential abundance estimates: the log_2_ difference in RPKM plotted as a function of transcript length (panel A) and with respect to P-value of Fisher’s Exact Test (panel B). Genes two standard deviations or more from the local mean are shaded in Figure 6A, which include 15 genes up-regulated with host infection and 28 that were down-regulated. All of these genes (Additional file 2: Table S2) had highly significant P-values by Fisher’s exact test and their annotation reveals a number of genes involved in the transport and metabolism of nutrients, particularly ions and sugars, which is consistent with a regulated response to changes in nutrient availability. In fact, the up-regulated gene with the highest P-value by Fisher’s exact test, AAPI14316, is homologous to the yeast glycerol/H + symporter STL1 [42], and the most significantly down-regulated gene, AAPI11676, is homologous to D-arabinitol dehydrogenase [43], an enzyme closely linked to glycerol metabolism. This concordant change in gene expression strongly suggests a shift in glycerol metabolism related to changing carbohydrate availability. Two additional genes with increased expression may be indicative of an increased metabolic rate during host infection: superoxide dismutase (AAPI10195) and SBDS (AAPI14490), which encodes a protein involved in ribosomal biogenesis and rRNA metabolism [44].

Three genes were up-regulated that are likely involved in the regulation of the plasma membrane and fungal cell wall, and thus could contribute to virulence. These genes were AAPI15600, a homolog of SUR7, a regulator of plasma membrane organization; AAPI12340, encoding a GTPase with a closest homolog in *S. cerevisiae* that is implicated in 1,3-alpha-glucan biosynthesis; and AAPI13585, encoding a non-secreted phospholipase A2. Another up-regulated gene (AAPI20003) is an 811-bp spliced transcript that is well supported by cDNA data but which is only predicted to encode a 62 amino acid peptide with no detected homology to GenBank proteins. It is possible that this is in fact a noncoding, regulatory transcript. All of these genes are useful candidates for further study by molecular methods such as *in situ* hybridization, quantitative PCR, or RNAi.

## Conclusions

From more than 1.5 million species that comprise the fungal kingdom, genomes of only about 82 species have been completed to date, but many more are currently in the pipeline. Among those sequenced, about 66 belong to Ascomycetes (http://www.yeastgenome.org/cgi-bin/blast-fungal.pl; http://www.broadinstitute.org). None of them are close relatives to the honey bee pathogen described in this study [45]. Indeed, while *A. apis* has been studied since the early 1950’s [1], genetic data for this pathogen were limited until very recently. Following the sequencing of the *A. apis* genome in 2006 [7], our comprehensive, transcriptome-informed gene annotations constitute a major step forward in delineating the molecular processes underlying its life history, including complex interactions with its host.

In this study we have provided a revised annotation of *A. apis* that incorporates extensive transcriptomic data, resulting in ~7,000 predicted protein-coding genes. We have annotated components of molecular pathways likely to contribute to virulence and reproduction. Following our previous description of the MAT-2 idiomorph [8], here we describe the second mating type allele (MAT-1) encoding α-box transcription factor, MAT1-1. We have also found other transcripts involved in the pheromone biosynthesis pathway, including pheromone receptors, binding proteins and efflux pumps, but failed to detect any sequences of the predicted fungal pheromones similar to those identified in genomes of heterothallic filamentous Ascomycetes. This could be due to the fact that these sequences are very short and highly diverged between Ascomycota. In support of this possibility, an extremely low degree of nucleotide conservation in these genes suggests a very rapid rate of evolution [38]. Alternatively, it is also possible that entirely different types of pheromones assumed a communication role in *A. apis*.

The differentially expressed genes identified here include candidate virulence factors that can be further investigated with molecular methods. Host pathogenesis is a series of events progressing from invasion and inhibition of host defenses, to massive proliferation of the fungus within the host. Every step of this process is well coordinated by activation of a wide range of virulence factors. Based on our findings, we propose an alternative strategy employed by *A. apis* during host invasion that differs from that suggested previously [27]. Our transcriptome analysis showed activation of a wide range of the hydrolytic enzymes that may help fungus to overcome host protective barriers. Specifically, transcripts encoding fungal chitinases may assist in penetration of the larval gut peritrophic membrane (PM) during host invasion and the cuticle at the final stage of exiting from a cadaver. Furthermore, we found components of the Aflotoxin-Sterigmatocystin (ST) biosynthesis pathway. Among those, *AflR* (AAPI16014) is a regulatory gene that controls transcription of the AF/ST pathway genes and *StcU/Ver1* (AAPI15087) was implicated in a critical step of toxin biosynthesis in *Aspergillus spp.*, the conversion of Versicolorin A to ST [46-48].

Collectively, our results provide new hypotheses and significant resources for investigating *A. apis* pathogenicity. Data generated in this study has been submitted to the public databases, the Bee Pests and Pathogens website and NCBI. It will become a platform shared by many scientists to decipher critical biochemical events occurring during host pathogenesis and expected to contribute ultimately to the control of the pathogen, improve honey bee health, and increase security for the worldwide food supply.

## Methods

### Production of fungal tissue

Mixed cultures of *A. apis* strains ARSEF 7405 and 7406 [7] were grown at 35° C in liquid or solid YGPS growth media [2] containing ampicillin (100 μg/ml) and streptomycin (12 μg/ml). Plate cultures were grown under 6% CO_2_ for 4 d, and liquid cultures were grown under normal atmosphere with shaking for 6 d. Plate cultures were visually inspected for contamination and liquid cultures were plated to check for contamination. Only uncontaminated cultures were further processed. Mycelia and spores were harvested and washed three times with ice-cold 0.9% NaCl, blotted dry, and stored at −80° C until used for total RNA isolation.

### Infection of honey bee larvae

Mixed cultures of ARSEF 7405 and 7406 [7] were grown at 35° C 6% CO_2_ on YGPS plates for two weeks, and spores were recovered from plate surfaces by rinsing with 0.001% Triton X100. The spore suspension was washed three times and resuspended in ice-cold dH_2_O to a final concentration that yielded a spore stock with an OD_600_ of 16. Plating confirmed that the suspension was free of contaminants. The spore stock was diluted 20-fold into 33° C mixed diet [49]. The infection of honey bee larvae was performed basically as described by [50]. Briefly, three-day-old larvae in groups of ten were fed with 500 μl of the spore suspension in mixed diet (~10 ^5^ spores per larva), and kept at 33° C under 97% RH in 6-well culture dishes. Subsequent feedings were done as needed using mixed diet without spores. Upon death of a larva, it was removed to a sterile Petri dish and incubation was continued under the same temperature and humidity conditions for 18–24 h. Larval remains were macerated and combined with an equal volume of ice cold 3% sodium citrate (pH 8.4) containing 5 μg/ml actinomycin D, and stored at −80° C. A small amount of the material from each sample was viewed under a microscope to confirm the presence of *A. apis* ascomata, and any sample lacking such spore cysts was discarded.

### RNA isolation

Frozen aliquots were rapidly ground in Trizol reagent (Invitrogen) using Teflon pestles in microcentrifuge tubes. RNA was subsequently isolated following the manufacturer’s instructions. DNA was removed from the preparations with DNAseI (Applied Biosystems, Austin TX) followed by the removal of rRNA with Ambion’s Poly (A) Purist kit (Applied Biosystems, Austin TX). The absence of *A. apis* and honey-bee DNA following DNAseI treatment was confirmed by PCR using actin species-specific primers (Table 2).

**Table 2:** List of primers used in this study

Primer name	Annealing temperature (°C)	Sequence
5′MAT2	58	CTGGGCGCAAATCTCCTG
DNALR1*	55	GTCATAGCAAAGGCAGT
MAT-2 F*	62	CGACAAAGCTGTGCATATC
MAT-2 R*	62	GGAGCATATTGGTAATTTGG
MAT-1 F	55	ATGTCAGCATTCGTGACAG
MAT-1 R	58	CATCGTTGGCTGAAAGAG
Actin_Aapis F	58	CATGATTGGTATGGGTCAG
Actin_Aapis R	60	CGTTGAAGGTCTCGAAGAC
Actin_Amel F	60	TGAAGTGCGATGTCGATATC
Actin_Amel R	60	GAGATCCACATCTGTTGGAA

*Described previously in Aronstein et al., 2007

### RNA quality and integrity control

Total RNA concentration was determined with a NanoDrop ND-1000 spectrophotometer (ThermoFisher Scientific) and brought to a 600 ng/μl concentration. RNA integrity was determined using an RNA 6000 Nano Chip and run on an Agilent 2100 Bioanalyzer (Agilent Technology Inc.). Next, an RNA Pico Chip was used to determine how well rRNA was removed and to visualize the size of the mRNA transcripts. Samples were diluted to 5 ng/μl using RNAse-free H_2_O, denatured for 2 min at 70° C then immediately placed on ice. One microliter of each sample was run on the RNA Pico Chip and the remainder stored at −80° C.

### cDNA library construction and whole transcriptome sequencing

Double-stranded cDNA was amplified with the cDNA Synthesis System (Roche Applied Science) using random hexamer priming, according to a manufacturer’s protocol. For each sample, 200 ng of cDNA was used to make a sequencing library using the GS FLX Titanium Rapid Library Preparation Kit Roche 454 Life Sciences). Each library was bar-coded with a unique 10-bp sequence. Each library was diluted to 1.0E7 molecules/μl in a 500 μl total volume and stored at −20 °C until sequencing. Samples were sequenced on a Roche 454 Titanium 200 Sequencer at the Purdue University Sequencing Center (Purdue University, Lafayette, IN). The output sequence was assembled by the Sequencing Center using GS Assembler (Roche 454 Life Sciences). Reads from axenic culture and host infection were assembled separately for transcript prediction with Maker (see below) as well as jointly for estimating the completeness of the genome.

### Gene prediction

The Maker annotation pipeline [9] was used to annotate the *A. apis* genome [7]. Maker masked repetitive sequences with RepeatMasker (http://www.repeatmasker.org) using a repeat library generated with RepeatModeler (version 1.0.3, http://www.repeatmasker.org/RepeatModeler.html). Maker used WU-BLAST [51] and Exonerate [52] to align EST and protein evidence to the genome. The assembled 454 isotig sequences from both the axenic culture and host infection samples were used as EST evidence, of which MAKER aligned 12,296 out of 16,191 isotigs (75.9%) to the genome scaffold assembly. Protein evidence included protein sequences from *A. apis* and four other fungal species (*A. capsulatusA. fumigatusC. immitis* and *Emericella nidulans*) downloaded from GenBank [53] in addition to all fungal proteins downloaded from UniProtKB/SwissProt [54].

Two *ab initio* gene predictors were trained for use with Maker: SNAP [55] and Augustus [56]. SNAP was trained in a bootstrap process as described below. Augustus was trained using the training pipeline packaged with the software (autoAug.pl) and the assembled 454 isotigs from both samples as evidence. Maker was run a total of four times. All of the EST and protein evidence was aligned to the scaffold sequences in the first run. These alignments were re-formatted as an initial training set for SNAP. Maker was then run a second and third time with SNAP as the gene predictor, re-training the SNAP prediction algorithm between each run using the gene annotations in order to improve the algorithm. Both SNAP and Augustus were used as gene predictors in the fourth run of MAKER to produce a final set of gene annotations, which included 6,718 genes encoding 6,771 transcripts.

The gene annotations produced by Maker were scanned with InterProScan [57] to identify protein domain signatures in 5,149 of the transcripts (76%) and assign GO identifiers. A set of 1,352 *ab initio* predictions that do not overlap these evidence-based annotations were also scanned with InterProScan. The 299 predictions (22.1%) that contained protein domain signatures were promoted to the gene set. Genes with repeat or viral associated protein domains were then removed from the gene set, resulting in a final official computational gene set containing 6,992 genes encoding 7,045 transcripts. Of these, 5,423 transcripts have at least one protein domain recognized by InterProScan. Putative functions were assigned to the annotations by using BLASTP [58] to identify homologs in the UniProt/SwissProt database (downloaded 07-22-2010).

All gene annotations and supporting evidence alignments produced using Maker along with protein domain signatures from InterProScan were loaded into a GBrowse genome browser available on the Bee Pests and Pathogens website (http://hymenopteragenome.org/beebase/?q=bee_pathogens). BLAST databases containing the scaffold assembly, the official and *ab initio* gene sets, NCBI GenBank predictions and manual annotations are also available.

### Phylogenetics

We estimated a species-level phylogeny of Eurotiomycete fungi based on sequences orthologous to the following *A. apis* protein predictions: dynein (AAPI10293), hexokinase (AAPI15521), RNA polymerase II beta subunit (AAPI13358), glycogen synthase (AAPI13950), Sec63 (AAPI11230), HMG-CoA reductase (AAPI14435), Not1 (AAPI11076), Orc2 (AAPI15426), Nmd3 (AAPI14923), chalcone synthase (AAPI11888), pantothenate synthase (AAPI12081), and TFIID subunit 9 (AAPI15764). For each additional species, the presumed ortholog was identified as a single, high-scoring BLASTP match that aligned across the breadth of the sequence. These species were *N. crassaMetarhizium acridumGibberella zeaeA. nidulansA. fumigatusPenicillium marnefeiiArthroderma otaeTrichophyton equinumParacoccidioides brasiliensisC. immitisC. posadasiiA. capsulatusA. dermatitidis*, and *U. reesii*[10,11,22,59,60]*.* Loci were aligned individually with MUSCLE [61] and then concatenated after deleting poorly aligned regions. MEGA5 [62] was used to determine the best fitting model of sequence evolution (JTT + G + I) and then to compute a maximum-likelihood tree with five rate categories and 1000 bootstrap replicates (other algorithms produced identical topologies).

### Manual annotation methods

Transcripts were compared to sequences in GenBank. TBLASTX and BLASTX searches were done against translated nucleotide databases and the non-redundant protein databases respectively. The deduced amino acid sequences were analyzed using the NCBI Conserved Domain Database (CCD) search service (version v2.14) and NCBI Conserved Domain Architecture Retrieval (CDART) tool. Manually annotated transcripts and the Bee Pests and Pathogens database accession (AAPI) numbers are provided in the Additional file 1: Table S1.

### Relative gene expression

Individual sequence reads were mapped with Mega BLAST [63] at a 97% identity threshold (read mapping with SSAHA2 [64] produced highly concordant results). Because the current genome assembly [7] includes regions of high similarity, the Maker-predicted transcripts were clustered with BLAST (97% identity and 70% overlap) prior to mapping to reduce redundancy in the reference. Gene expression estimates were considered both as raw counts and normalized to transcript length (RPKM). We considered genes to be strong candidates for having differential expression between axenic culture and host infection if 1) the log_2_ difference in RPKM was greater than two standard deviations from the mean, and 2) if the read counts were significantly different by Fisher’s Exact Test with Benjamini-Hochberg correction for multiple tests, calculated with the edgeR package [41]. Because the variance in read counts is in part a function of transcript length [65], we calculated the standard deviation in differential expression as a sliding window of the 500 closest genes in terms of transcript length. The variance in expression is also a function of expression level, but we did not attempt to correct for this because expression is expected to be highly context dependent for biological reasons whereas transcript length is not (disregarding alternative splicing). This *ad hoc* method is intentionally conservative because no replication of the sequence library is available, and thus a variance estimate for individual transcripts is not possible. We emphasize that replicated experiments are needed to confirm that these genes are differentially expressed under each growth condition.

### Amplification of the MAT1-1 gene from the α-box idiomorph

After 6 days of *A. apis* growth on YPGS culture medium, genomic DNA was isolated with a phenol/chlorophorm method as described in [5]. Three sets of primers were used to amplify the portions of the MAT locus from MAT-1 and MAT-2 strains (Table 2). PCR assays were conducted with a PTC-200 automated thermal cycler (MJ Research). Genomic DNA was amplified in a 30-μl reaction mixture using the following amplification conditions: a 98 °C (30 s) step, 30 cycles of 98 °C (10 s), 58 °C (30 s), and 72 °C (3 min or 1 min) step. PCR products were visualized by 0.8% agarose gel electrophoresis in 1 × TAE buffer (40 mM Tris-acetate,1 mM EDTA) and ethidium bromide (0.2 μg/mL) staining.

## Authors’ contributions

RSC and AKB carried out data analysis, conducted interpretations of the results and contributed to manuscript; JDE and CGE reviewed early drafts of the manuscript and contributed immensely to its improvement; KDM participated in pathogen related work and drafted the manuscript; KA contributed to the conception and design of the study, conducted manual gene annotations and wrote the initial version of the manuscript. All authors read and approved of the final manuscript.

## Supplementary Material

Additional file 1 **Table S1. (Supplemental).*A. apis*manual gene annotation according to functional groups**. Showing gene names, database (GenBank or Bee Pests and Pathogens/AAPI) accession numbers and names of species with the highest similarity. If sequences were not found in either of the two databases, they were referenced by the isotig or contig trace numbers. Datasets referenced in this table are available as follows: Baylor College of Medicine Genome Project 17285, *Ascosphaera apis* USDA-ARSEF 7405 and Bee Pests and Pathogens (http://hymenopteragenome.org/beebase/?q=bee_pathogens). Click here for file

Additional file 2 **Table S2. (Supplemental). Genes that are strong candidates for differential expression between axenic culture and host infection**. Genes listed have both a length-normalized expression differential greater than two standard deviations from the local mean (see text) and a significant result of Fisher’s Exact Test for equal abundance of reads (with Benjamini-Hochberg correction for multiple tests). Click here for file

## References

[B1] Spiltoir CF Life cycle of Ascosphaera apis Am J Bot 1955 42 501 518 10.2307/2438686

[B2] Bailey L Ball BV Honey Bee Pathology 1991 Academic, London, UK

[B3] Gilliam M Vandenberg JD Morse R, Flottum K Fungi Honey Bee Pests, Predators, and Diseases 1997 AI Root, Ohio, OH 81 110

[B4] Heath LAF Occurrence and distribution of chalk brood disease of honeybees Bee World 1985 66 9 15

[B5] Aronstein K Murray KD Chalkbrood disease in honey bees J Invertebr Pathol 2010 103 20 29 10.1016/j.jip.2009.06.018 19909969

[B6] Hornitzky M Literature review of chalkbrood. A report for the RIRDC 2001 ACT, AU, Kingston

[B7] Qin X Evans JD Aronstein K Murray KD Weinstock GM Genome sequences of the honey bee pathogens Paenibacillus larvae and Ascosphaera apis Insect Mol Biol 2006 15 715 718 10.1111/j.1365-2583.2006.00694.x 17069642 PMC1761131

[B8] Aronstein KA Murray KD de Leon J Qin X Weinstock G High mobility group (HMG-box) genes in the honey bee fungal pathogen Ascosphaera apis Mycologia 2007 99 553 561 10.3852/mycologia.99.4.553 18065006

[B9] Cantarel BL Korf I Robb SMC Parra G Ross E Moore B Holt C Alvarado AS Yandell M MAKER: an easy-to-use annotation pipeline designed for emerging model organism genomes Genome Res 2008 18 188 196 18025269 10.1101/gr.6743907 PMC2134774

[B10] Galagan JE Calvo SE Cuomo C Ma L-J Wortman JR Batzoglou S Lee S-I Batürkmen M Spevak CC Clutterbuck J Sequencing of Aspergillus nidulans and comparative analysis with A. fumigatus and A. oryzae Nature 2005 438 1105 1115 10.1038/nature04341 16372000

[B11] Galagan JE Calvo SE Borkovich KA Selker EU Read ND Jaffe D FitzHugh W Ma L-J Smirnov S Purcell S The genome sequence of the filamentous fungus Neurospora crassa Nature 2003 422 859 868 10.1038/nature01554 12712197

[B12] Stein LD Mungall C Shu S Caudy M Mangone M Day A Nickerson E Stajich JE Harris TW Arva A Lewis S The generic genome browser: a building block for a model organism system database Genome Res 2002 12 1599 610 10.1101/gr.403602 12368253 PMC187535

[B13] Munoz-Torres MC Reese JT Childers CP Bennett AK Sundaram JP Childs KL Anzola JM Milshina NV Elsik CG Hymenoptera Genome Database: integrated community resources for insect species of the order Hymenoptera Nucleic Acids Res 2011 39 D658 D662 10.1093/nar/gkq1145 21071397 PMC3013718

[B14] Wang B Guo G Wang C Lin Y Wang X Zhao M Guo Y He M Zhang Y Pan L Survey of the transcriptome of Aspergillus oryzae via massively parallel mRNA sequencing Nucleic Acids Res 2010 38 5075 5087 10.1093/nar/gkq256 20392818 PMC2926611

[B15] Parra G Bradnam K Ning Z Keane T Korf I Assessing the gene space in draft genomes Nucleic Acids Res 2009 37 298 297 10.1093/nar/gkn925 19042974 PMC2615622

[B16] Geiser DM Gueidan C Miadlikowska J Lutzoni F Kauff F Hofstetter V Fraker E Schoch CL Tibell L Untereiner WA Aptroot A Eurotiomycetes: Eurotiomycetidae and Chaetothyriomycetidae Mycologia 2006 98 1053 1064 10.3852/mycologia.98.6.1053 17486980

[B17] Eddy SR Profile hidden Markov models Bioinformatics 1998 14 755 763 10.1093/bioinformatics/14.9.755 9918945

[B18] Eddy SR Mitchison G Durbin R Maximum discrimination hidden Markov models of sequence consensus J Comp Biol 1995 2 9 23 10.1089/cmb.1995.2.9 7497123

[B19] Petersen TN Brunak S Heijne Gv Nielsen H SignalP 4.0: discriminating signal peptides from transmembrane regions Nat Methods 2011 8 785 786 10.1038/nmeth.1701 21959131

[B20] Krogh A Larsson B Heijne Gv Sonnhammer ELL Predicting Transmembrane Protein Topology with a Hidden Markov Model: Application to Complete Genomes J Mol Biol 2001 305 567 580 10.1006/jmbi.2000.4315 11152613

[B21] Wu C Kim Y-S Smith KM Li W Hood HM Staben C Selker EU Sachs MS Farman ML Characterization of Chromosome Ends in the Filamentous Fungus Neurospora crassa Genetics 2009 181 1129 1145 10.1534/genetics.107.084392 19104079 PMC2651048

[B22] Sharpton TJ Stajich JE Rounsley SD Gardner MJ Wortman JR Jordar VS Maiti R Kodira CD Neafsey DE Zeng Q Comparative genomic analyses of the human fungal pathogens Coccidioides and their relatives Genome Res 2009 19 1722 1731 10.1101/gr.087551.108 19717792 PMC2765278

[B23] Ahn J-H Cheng Y-Q Walton JD An Extended Physical Map of the TOX2 Locus of Cochliobolus carbonum Required for Biosynthesis of HC-Toxin Fungal Genet Biol 2002 35 31 38 10.1006/fgbi.2001.1305 11860263

[B24] Reichard U Cole GT Rüchel R Monod M Molecular cloning and targeted deletion of PEP2 which encodes a novel aspartic proteinase from Aspergillus fumigatus Int J Med Microbiol 2000 290 85 96 10.1016/S1438-4221(00)80111-3 11043985

[B25] Duttaa K Sen S Veerank VD Production, characterization and applications of microbial cutinases Process Biochem 2009 44 127 134 10.1016/j.procbio.2008.09.008

[B26] Raynaud C Guilhot C Rauzier J Bordat Y Pelicic V Manganelli R Smith I Gicquel B Jackson M Phospholipases C are involved in the virulence of Mycobacterium tuberculosis Mol Microbiol 2002 45 203 217 10.1046/j.1365-2958.2002.03009.x 12100560

[B27] Alonso JM Rey J Puerta F Hermoso de Mendoza J Hermoso de Mendoza M Flores JM Enzymatic equipment of Ascosphaera apis and the development of infection by this fungus in Apis mellifera Apidologie 1993 24 383 390 10.1051/apido:19930404

[B28] Theantana T Chantawannakul P Protease and ß-N acetylglucosaminidase of honey bee chalkbrood pathogen Ascosphaera apis J Apic Res 2008 47 68 76

[B29] Maghrabi HA Kish LP Isozyme characterization of Ascocphaerales associated with bees. Ascosphaera apis, Ascosphaera proliperda, and Ascosphaera aggregata Mycologia 1985 77 358 365 10.2307/3793191

[B30] Heath LAF Chalk brood pathogens: a review Bee World 1982 63 130 135

[B31] Chmielewski M Glinski Z Studies on pathogenicity of Ascosphaera apis for larvae of the honeybee (Apis mellífera L.). Part I. Biochemical propierties of A. apis Ann Univ Mariae Curie-Sklodowska 1981 36 71 82

[B32] Kowalska M Biochenical properties of Ascosphaera apis and Bettsia alvei Polsk Arch Wet 1984 24 7 15

[B33] Henrissat B A classification of glycosyl hydrolases based on amino acid sequence similarities Biochem J 1991 280 309 316 1747104 10.1042/bj2800309 PMC1130547

[B34] Cogoni C Macino G Gene silencing in Neurospora crassa requires a protein homologous to RNA-dependent RNA polymerase Nature 1999 399 166 169 10.1038/20215 10335848

[B35] Hammond TM Bok JW Andrewski MD Reyes-Domínguez Y Scazzocchio C Keller NP RNA Silencing Gene Truncation in the Filamentous Fungus Aspergillus nidulans Eukaryot Cell 2008 7 339 349 10.1128/EC.00355-07 18065653 PMC2238149

[B36] Aronstein KA Oppert B Lorenzen MD Grabowski P RNAi in the agriculturally important arthropods, in RNA Processing RNA Processing 2011 InTech, Rijeka, Croatia 157 180

[B37] Kronstad JW Staben C Mating type in filamentous fungi Annu Rev Genet 1997 31 245 276 10.1146/annurev.genet.31.1.245 9442896

[B38] Poggeler S Mating-type genes for classical strain improvements of Ascomycetes Appl Microbiol Biotechnol 2001 56 589 601 10.1007/s002530100721 11601605

[B39] Cozijnsen AJ Howlett BJ Characterization of the mating-type locus of the plant pathogenic ascomycete Leptosphaeria maculans Curr Genet 2003 43 351 357 10.1007/s00294-003-0391-6 12679880

[B40] Rau D Maier FJ Papa R Brown AH Balmas V Saba E Schaefer W Attene G Isolation and characterization of the mating-type locus of the barley pathogen Pyrenophora teres and frequencies of mating-type idiomorphs within and among fungal populations collected from barley landraces Genome 2005 48 855 869 10.1139/g05-046 16391692

[B41] Robinson MD McCarthy DJ Smyth GK edgeR: a Bioconductor package for differential expression analysis of digital gene expression data Bioinformatics 2010 26 139 140 10.1093/bioinformatics/btp616 19910308 PMC2796818

[B42] Ferreira C Voorst Fv Martins A Neves L Oliveira R Kielland-Brandt MC Lucas C Brandt A A Member of the Sugar Transporter Family, Stl1p Is the Glycerol/H+ Symporter in Saccharomyces cerevisiae Mol Biol Cell 2005 16 2068 2076 10.1091/mbc.E04-10-0884 15703210 PMC1073684

[B43] Hallborn J Walfridsson M Penttila M Keranen S Hahn-Hagerdal B A short-chain dehydrogenase gene from Pichia stipitis having D-arabinitol dehydrogenase activity Yeast 1995 11 839 847 10.1002/yea.320110906 7483848

[B44] Savchenko A Krogan N Cort JR Evdokimova E Lew JM Yee AA Sanchez-Pulido L Andrade MA Bochkarev A Watson JD The Shwachman-Bodian-Diamond Syndrome Protein Family Is Involved in RNA Metabolism J Biol Chem 2005 280 19213 19220 10.1074/jbc.M414421200 15701634

[B45] Sugiyama M Ohara A Mikawa T Molecular phylogeny of onygenalean fungi based on small subunit ribosomal DNA (SSU rDNA) sequences Mycoscience 1999 40 251 258 10.1007/BF02463962

[B46] Brown DW Yu J-H Kelkar HS Fernandes M Nesbitt TC Keller NP Adams TH Leonard TJ Twenty-five coregulated transcripts define a sterigmatocystin gene cluster in Aspergillus nidulans Proc Natl Acad Sci USA 1996 93 1418 1422 10.1073/pnas.93.4.1418 8643646 PMC39953

[B47] Keller NP Segner S Bhatnagar D Adams TH stcS, a Putative P-450 Monooxygenase, Is Required for the Conversion of Versicolorin A to Sterigmatocystin in Aspergillus nidulans Appl Environ Microbiol 1995 61 3628 3632 7486998 10.1128/aem.61.10.3628-3632.1995 PMC167660

[B48] Cary JW Ehrlich KC Aflatoxigenicity in Aspergillus: molecular genetics, phylogenetic relationships and evolutionary implications Mycopathologia 2006 162 167 177 10.1007/s11046-006-0051-8 16944284

[B49] Aronstein KA Murray KD Saldivar E Transcriptional responses in honey bee larvae infected with chalkbrood fungus BMC genomics 2010 11 2 10.1186/1471-2164-11-2 20565973 PMC2996924

[B50] Aronstein K Saldivar E Characterization of a honey bee Toll related receptor gene Am18w and its potential involvement in antimicrobial immune defense Apidologie 2005 36 3 14 10.1051/apido:2004062

[B51] Korf I Yandell M Bedell J BLAST 2003 O’Reilly Media, Sebastopol, CA 368

[B52] Slater GS Birney E Automated generation of heuristics for biological sequence comparison BMC Bioinforma 2005 6 31 10.1186/1471-2105-6-31 PMC553969 15713233

[B53] Benson DA Karsch-Mizrachi I Clark K Lipman DJ Ostell J Sayers EW GenBank Nucleic Acids Res 2012 40 D48 D53 10.1093/nar/gkr1202 22144687 PMC3245039

[B54] Consortium U The Universal Protein Resource (UniProt) in 2010 Nucleic Acids Res 2010 38 Database issue D142 148 19843607 10.1093/nar/gkp846 PMC2808944

[B55] Korf I Gene finding in novel genomes BMC Bioinforma 2004 5 10.1186/1471-2105-5-59 PMC421630 15144565

[B56] Stanke M Tzvetkova A Morgenstern B AUGUSTUS at EGASP: using EST, protein and genomic alignments for improved gene prediction in the human genome Genome Biol 2006 7 S11 11 18 10.1186/gb-2006-7-s1-s11 PMC1810548 16925833

[B57] Zdobnov EM Apweiler R InterProScan-an integration platform for the signature-recognition methods in InterPro Bioinformatics 2001 17 847 848 10.1093/bioinformatics/17.9.847 11590104

[B58] Altschul SF Gish W Miller W Meyers EW Lipman DJ Basic Local Alignment Search Tool J Mol Biol 1990 215 403 410 2231712 10.1016/S0022-2836(05)80360-2

[B59] Gao Q Jin K Ying S-H Zhang Y Xiao G Shang Y Duan Z Hu X Xie X-Q Zhou G Genome Sequencing and Comparative Transcriptomics of the Model Entomopathogenic Fungi Metarhizium anisopliae and M. acridum PLoS Genet 2011 7 e1001264 10.1371/journal.pgen.1001264 21253567 PMC3017113

[B60] Cuomo CA Güldener U Xu J-R Trail F Turgeon BG Pietro AD Walton JD Ma L-J Baker SE Rep M The Fusarium graminearum Genome Reveals a Link Between Localized Polymorphism and Pathogen Specialization Science 2007 317 1400 1402 10.1126/science.1143708 17823352

[B61] Edgar RC MUSCLE: multiple sequence alignment with high accuracy and high throughput Nucleic Acids Res 2004 32 1792 1797 10.1093/nar/gkh340 15034147 PMC390337

[B62] Tamura K Peterson D Peterson N Stecher G Nei M Kumar S MEGA5: Molecular Evolutionary Genetics Analysis using Maximum Likelihood, Evolutionary Distance, and Maximum Parsimony Methods Mol Biol Evol 2011 10 2731 2739 21546353 10.1093/molbev/msr121 PMC3203626

[B63] Zhang Z Schwartz S Wagner L Miller W Agreedy algorithm for aligning DNAsequences J Comp Biol 2000 7 203 214 10.1089/10665270050081478 10890397

[B64] Ning Z Cox AJ Mullikin JC A fast search method for large DNA databases Genome Res 2001 11 1725 1729 10.1101/gr.194201 11591649 PMC311141

[B65] Oshlack A Wakefield MJ Transcript length bias in RNA-seq data confounds systems biology Biol Direct 2009 4 14 10.1186/1745-6150-4-14 19371405 PMC2678084

