# Supplementary material for: Transcriptome analysis of the honey bee fungal pathogen, Ascosphaera apis: implications for host pathogenesis
Source: BMC Genomics. 2012 Jun 29;13:285. doi: 10.1186/1471-2164-13-285 (PMC3425160; doi:10.1186/1471-2164-13-285)
Supplement: Additional file 1 — Table S1. (Supplemental).A. apismanual gene annotation according to functional groups. Showing gene names, database (GenBank or Bee Pests and Pathogens/AAPI) accession numbers and names of species with the highest similarity. If sequences were not found in either of the two databases, they were referenced by the isotig or contig trace numbers. Datasets referenced in this table are available as follows: Baylor College of Medicine Genome Project 17285, Ascosphaera apis USDA-ARSEF 7405 and Bee Pests and Pathogens (http://hymenopteragenome.org/beebase/?q=bee_pathogens). [file 1471-2164-13-285-S1.doc]

Table S1 Supplemental. *A. apis* genome analysis according to functional groups:

| **Gene Name** | ***A. apis* gene predictions** | **Gene name and function** | **Notes & Similarity** |
| --- | --- | --- | --- |
| **Pheromone response** | | | |
| *Mat1-1* | AapisI_contig04567_3; AapisC_contig01774_1 | Alpha mating-type transcription factor | *Ajellomyces capsulatus* |
| *Mat1-2* | GenBank: ABN1478.1 | HMG-box DNA-binding proteins | *Ajellomyces capsulatus* |
| *Sla2* | AAPI11102 | Cytoskeleton assembly control protein Sla2 | *Ajellomyces capsulatus*  Flanks MAT 2 locus |
| *Rim-2* | AAPI11104 | Mitochondrial carrier protein | *Saccharomyces cerevisiae*  Flanks MAT 2 locus |
| *Apn2* | AAPI11089 | DNA lyase, repair protein | *Aspergillus fumigatus* Flanks MAT 2 locus |
| *Swi10* | AAPI15761 | Mating-type switching protein | *Ajellomyces dermatitidis* |
| *Htf* | AAPI40003 | HMG-box transcription factor | *Ajellomyces capsulatus* |
| *Ste2/PreB* | AAPI40023 | Alpha mating type pheromone receptor | *Aspergillus fumigatus* |
| *Ste4/Prp4* | AAPI10745 | STE4 beta subunit of G protein coupled to pheromone receptor | *Saccharomyces cerevisiae* |
| *Mcm1* | AAPI12563 | MADS-box pheromone receptor transcription factor | *Saccharomyces cerevisiae* |
| *Ste23* | AAPI12150 | Alpha pheromone processing metallopeptidase | *Aspergillus fumigatus* |
| *ProA* | AAPI14209 | Gamma-glutamyl phosphate reductase, regulation of sexual sporulation | *Aspergillus fumigatus* |
| *DapB* | AapisC_contig05758; AapisI_contig03828 | Pheromone maturation dipeptidyl aminopeptidase | *Neosartorya fischeri* |
| *Ste6* | AAPI13966 | Alpha-mating factor transporting ATPase | *Saccharomyces cerevisiae* |
| *Ste11* | AAPI30240 | HMG-box transcription factor | *Ajellomyces capsulatus* |
| *STE-50* | AAPI11510 | Adaptor protein regulate mating response and invasive filamentous growth | *Saccharomyces pombe* |
| *ApidoR1* | AAPI14096 | Apidonectin receptor | *Homo sapiens* |
| *AtrD* | AapisI_contig03224; AapisC_contig05899 | ABC a-pheromone efflux pump | *Aspergillus fumigatus* |
| *Far11* | AapisI_contig04785; AapisC_contig03724 | Pheromone-dependent cell cycle arrest protein | *Aspergillus clavatus* |
| *Kex1* | AapisC_contig06851; AapisC_contig01301 | Pheromone processing carboxypeptidase | *Aspergillus clavatus* |
| *PreA* | AapisC_contig06064 | Alpha-pheromone receptor | *Neosartorya fischeri* |
| Pheromone receptor | AapisC_isotig0517 | Pheromone-regulated membrane protein | *Aspergillus fumigatus* |
| Pheromone pump | AapisC_isotig05998 | Alpha-pheromone efflux pump | *Aspergillus fumigatus* |
| *FlbD* | AapisI_contig02302_4; AapisI_contig00679_4 | MYB family conidiophore developing protein | *Neurosartorya fischeri* |
| **Transcriptional and translation regulation** | | | |
| HMG-box | AAPI10586 | High mobility group protein B | *Coccidioides immitis* |
| *Ste12/SteA* | AAPI12324 | Homeodomain transcription factor, key regulator of fungal virulence | *Emericella nidulans* |
| *CreA* | AAPI12889; AAPI12890; AAPI10561; AAPI13364 | DNA-binding protein | *Aspergillus aculeatus* |
| *Mei2* | AAPI15864 | Meiosis protein, RNA binding | *Saccharomyces pombe* |
| *Taf2* | AAPI11394 | Transcription initiation factor TFIID subunit 2 | *Saccharomyces pombe* |
| *Taf3* | AAPI13940 | Transcription initiation factor TFIID subunit 3 | *Mus musculus* |
| *Tfc7* | AAPI12003 | Transcription factor tau subunit, a component of the TFIIIC complex | *Saccharomyces cerevisiae* |
| *Yap3* | AAPI12461 | AP-1 transcription factor | *Saccharomyces cerevisiae* |
| *Pap-1* | AAPI15841 | AP-1 transcription factor |  |
| *Swi5* | AAPI14423 | Transcription factor | *Saccharomyces cerevisiae* |
| *Ace1* | AAPI11924 | Zinc finger transcription factor | *Trichoderma reesei* |
| *Seb1* | AAPI13871 | Rpd7-binding protein, transcription factor | *Saccharomyces pombe* |
| *Cyp1* | AAPI14886  AAPI13379 | Peptidyl-prolyl isomerase-1, cyclophilin family, protein folding | *Aspergillus fumigatus* Target cite of drug cyclopsporin |
| *Cyp3* | AAPI14473  AAPI10236 | Peptidyl-prolyl isomerase H, cyclophilin family isomerase, protein folding | Target cite of drug cyclopsporin |
| *Cyp6* | AAPI10588 | Peptidyl-prolyl isomerase-4, cyclophilin family, protein folding | Target cite of drug cyclopsporin |
| *Cyp8* | AAPI13512 | Peptidyl-prolyl isomerase-2, cyclophilin family, protein folding | Target cite of drug cyclopsporin |
| *Cyp10* | AAPI14655 | Peptidyl-prolyl isomerase-3, cyclophilin family, protein folding | Target cite of drug cyclopsporin |
| *Cyp15* | AAPI13402 | Peptidyl-prolyl isomerase 15, cyclophilin family, protein folding | Target cite of drug cyclopsporin |
| *Sir2* | AAPI12180 | NAD-dependent histone deacetylase, transcriptional DNA silencing | *Saccharomyces cerevisiae* |
| *Irs4* | AAPI15438 | Increased rDNA silencing protein 4, transcriptional DNA silencing | *Aspergillus clavatus* |
| *Ef-1 alpha* | AAPI15403 | Elongation factor-1 gamma, GTP-binding protein | *Homo sapiens* |
| **MAPK signaling** | | | |
| *Win 1* | AAPI10652 | Stress activated MAP kinase kinase kinase | *Saccharomyces pombe* |
| *Mkk1/Ssp32* | AAPI13102 | MAP kinase kinase | *Saccharomyces cerevisiae* |
| *Mapkbp1* | AAPI10615 | MAP kinase-binding protein | *Homo sapiens* |
| *Hog1* | AAPI30120; AAPI10585 | MAP kinase | *Coccidioides immitis* |
| *Mapk* | AAPI10924; AAPI10925 | MAP kinase, invasive filamentous growth | *Fusarium solani* |
| *Spm1* | AAPI10090 | MAP kinase, respond to extracellular stimuli | *Saccharomyces pombe* |
| *Ste-11* | AAPI10558; AAPI15631 | Serine/threonine-protein kinase, cell-type-specific transcription | *Ajellomyces capsulatus* |
| *Pap-1* | AAPI15841 | AP-1 transcription factor regulated by MAPK pathways | *Saccharomyces pombe* |
| **RNAi pathway** | | | |
| *Dcl-2* | AAPI13688 | Dicer like protein 2, dsRNA-specific RNase-III type ribonuclease | *Coccidioides immitis* |
| *Dcl-1* | AAPI10297; AAPI10158 | Dicer like protein 1, dsRNA-specific RNase-III type ribonuclease | *Aspergillus terreus* |
| *Rbm25* | AAPI15628 | dsRNA-binding motif proteins (dsRBM), splicing factor, RNA metabolism | *Homo sapiens* |
| *Rbm5-a* | AAPI14493 | dsRNA-binding motif proteins (dsRBM), splicing factor, RNA metabolism | *Xenopus laevis* |
| *Ago1* | AAPI13326; AAPI10219 | RNase H enzyme Argonaute (AGO1) | *Saccharomyces pombe* |
| *Rdr1* | AAPI11103 | RNA-dependent RNA polymerase 1 (RdRP), RNA replicas | *Saccharomyces pombe* |
| *Sas10* | AAPI16445 | Something about silencing protein 10, gene silencing | *Saccharomyces pombe* |
| *Snd1* | AAPI15247; AAPI11773 | Nuclease domain containing 1/ p100 coactivator/ Tudor-SN, RISC nuclease | *Saccharomyces pombe* |
| *Rsd1* | AAPI11970; AAPI13350 | RNA binding proteins (RBPs), RNA metabolism, transport of silencing information | *Saccharomyces pombe* |
| **Environmental Stress** | | | |
| *Mak3* | AAPI12580 | Peroxide stress-activated histidine kinase | *Saccharomyces pombe* |
| *Mak1* | AAPI12577 | Peroxide stress-activated histidine kinase | *Saccharomyces pombe* |
| *yhdN* | AAPI13693 | General stress protein | *Saccharomyces pombe* |
| *Nst1* | AAPI11337; AAPI14604; AAPI14602; AAPI13092 | Stress response protein | *Bacillus subtilis* |
| *Ish1* | AAPI14614 | Stress response protein | *Aspergillus niger* |
| Stress protein | AAPI15942; AAPI14129 | Universal stress protein A family C25B2 | *Saccharomyces pombe* |
| *Cyt1* | AAPI11648 | Cytochrome c1 | *Saccharomyces pombe* |
| *Cyp52A1* | AAPI12100 | Cytochrome P450 52A1 | *Saccharomyces pombe* |
| *Cyp1A1* | AAPI14514 | Cytochrome P450 1A1 | Candida tropicalis |
| *Cyp61* | AAPI15696 | Cytochrome P450 61 | Opsanus tau |
| *Cyp110* | AAPI10626 | Cytochrome P450 110 | *Anabaena sp* |
| *Cox4* | AAPI15803 | Cytochrome c oxidase polypeptidase 5 | *Sacharomyces cerevisiae* |
| *Cox 5* | AAPI14791 | Cytochrome c oxidase polypeptidase 5 | *Sacharomyces cerevisiae* |
| *Cox13* | AAPI11269 | Cytochrome c oxidase subunit 6A | *Sacharomyces cerevisiae* |
| *Cox15* | AAPI12597 | Cytochrome c oxidase assembly protein | *Sacharomyces cerevisiae* |
| *Cox16* | AAPI10929 | Cytochrome c oxidase assembly protein | *Sacharomyces cerevisiae* |
| *Cox17* | AAPI14963 | Cytochrome c oxidase copper chaperone | *Sacharomyces cerevisiae* |
| *Cox19* | AAPI15920 | Cytochrome c oxidase assembly protein | *Sacharomyces cerevisiae* |
| *Cyp b5* | AAPI13048; AAPI15235; AAPI15233; AAPI11454; AAPI16394 | Cytochrome b5 | *Sacharomyces cerevisiae* |
| *Cyp b* | AAPI14649 | Cytochrome b | *Neurospora crassa* |
| *Cyp b2* | AAPI15996;AAPI15091; AAPI16467; AAPI12535 | Cytochrome b2, [oxidoreductase](http://www.google.com/url?sa=t&source=web&cd=4&ved=0CCkQFjAD&url=http%3A%2F%2Fwww.ncbi.nlm.nih.gov%2Fpmc%2Farticles%2FPMC554653%2Fpdf%2Femboj00277-0219.pdf&ei=dgHDTcCUL4rDgQfikeHdAQ&usg=AFQjCNGF1rLS4CwHdi0P8Vy6j2mqV_0HYA) | *Emericella nidulans* |
| *Cyp b5R4* | AAPI12445 | Cytochrome b5 reductase 4 | *Emericella nidulans* |
| *Qcr-2* | AAPI13870 | Cytochrome b-c1 complex subunit 2 | *Danio rerio* |
| *Qcr-6* | AAPI13857 | Cytochrome b-c1 complex subunit 6 | *Danio rerio* |
| *Cne* | AAPI14613 | Calnexin, ER stress-induced apoptosis | *Coccidioides immitis* |
| *Hsf1* | AAPI13959 | Heat shock factor protein | *Saccharomyces* *pombe* |
| *Hsp78* | AAPI11046; AAPI11045; AAPI14497 | Heat shock protein | *Saccharomyces pombe* |
| *Hsp70* | AAPI14978; AAPI11317; AAPI13471; AAPI12113 | Heat shock protein | *Emericella nidulans* |
| *Hsp10* | AAPI12025 | Heat shock protein | *Emericella nidulans* |
| *Hsp30* | AAPI13591; AAPI10004; AAPI12792 | Heat shock protein | *Emericella nidulans* |
| *Hsp88* | AAPI16209 | Heat shock protein | *Neurospora crassa* |
| *Hsp98* | AAPI11325 | Heat shock protein | *Neurospora crassa* |
| *Sti1* | AAPI14702; AAPI14919; AAPI30180 | Heat shock protein | *Saccharomyces* *pombe* |
| *Hsp60* | AAPI14788; AAPI14789 | Heat shock protein | *Saccharomyces* *pombe* |
| *Rci* | AapisI_contig06657_1; AapisI_contig06656_1 | Stress response RCI peptide | *Neosartorya fischeri* |
| **Virulence factors** | | | |
| *Csp* | AAPI11445 | Circumsporozoite protein  major parasite surface protein | *Plasmodium falciparum* |
| *Gp1* | AAPI11885; AAPI10589 | Vegetative cell wall protein | Highly immunogenic |
| *StcU* | AAPI15087 | Versicolorin A ketoreductase, 3-ketoacyl-acyl reductase, mycotoxin biosynthesis | *Penicillium marneffei*  *Aspergillus fumigatus* |
| *StcW* | AAPI14557 | Sterigmatocystin biosynthesis monooxigenase | *Emericella nidulans* |
| *OmtA* | AAPI10160; AAPI16494; AAPI16495; AAPI30135 | Sterigmatocystin 8-O-methiltransferase | *Emericella nidulans* |
| *OrdA* | AAPI13290 | O-methylsterigmatocystin oxidoreductase | *Aspergillus flavus* |
| *AflR* | AAPI16014 | Sterigmatocystin biosynthesis regulatory protein | *Aspergillus flavus* |
| *Syb1* | AAPI10875; AAPI10876 | Synaptobrevin homolog 1 | *Emericella nidulans* |
| *Ykt6* | AAPI10529 | Synaptobrevin homolog | *Saccharomyces pombe* |
| *Ski3* | AAPI15770 | Super killer protein 3 | *Saccharomyces pombe* |
| *Cts1* | AAPI10067; AAPI14422 | Endochitinase 1 class V, glycoside hydrolase family 18, chitin hydrolysis | *Saccharomyces pombe* |
| *Cts42* | AAPI12087 | Endochitinase class V, glycoside hydrolase family 18, chitin hydrolysis | *Coccidioides immitis* |
| *ChiA1* | AAPI13302 | Class III chitinase, GH18 | *Trichoderma harzianum* |
| *FabG* | AAPI14860; AAPI13982 | 3-oxoacyl reductase, fatty acid biosynthesis | *Aspergillus fumigatus* |
| *FabF* | AAPI13995 | Acyl synthase 2, fatty acid biosynthesis | *Bacillus subtilis* Target for drug Helicocerin |
| *ToxA* | AAPI13322; AAPI10692 | HC-toxin efflux carrier | *Rhizobium meliloti* Target for drug Helicocerin |
| *ToxG* | AAPI14773; AAPI15541 | Alanine racemase, HC-toxin biosynthesis | *Cochlibolus carbonum* |
| *ToxD* | AAPI16134 | Dehydrogenase, HC-toxin biosynthesis | *Cochliobolus carbonum* |
| *ToxF* | AAPI11585 | Branched chain amino acid transaminase, HC-toxin synthesis | *Cochlibolus carbonum* |
| *Hts1* | AAPI11407; AAPI12079 | Nonribosomal peptide synthetase, HC-toxin synthetase | *Cochlibolus carbonum* |
| *Nor-1* | AAPI11758; AAPI30214 | Aflotoxin biosynthesis ketoreductase | *Cochlibolus carbonum* |
| *Rac1* | AAPI12571 | Ras-related C3 botulinum toxin substrate 1 | *Aspergillus parasiticus* |
| *Ctf1-Alpha* | AAPI12114; AAPI14517; AAPI10786 | Cutinase transcription factor 1 alpha | *Bos taurus* |
| *Ctf1-Beta* | AAPI15257; AAPI11327 | Cutinase transcription factor 1 beta | *Fusarium solani* |
| *GlaA* | AAPI11641 | Extracellular glucoamylase | *Fusarium solani* |
| *Alta7* | AAPI127942 | Minor allergen, contains 1 wrdA family flavodoxin-like domain | *Alternaria alternata* |
| **Amidases** | | | |
| *Hex1* | AAPI15949 | Beta-hexosamidase | *Candida albicans* |
| Amidase | AAPI10680 | Amidase | *Candida albicans* |
| *Acer3* | AAPI12494 | Alkaline ceramidase | *Saccharomyces pomber* |
| *AmdS* | AAPI15265; AAPI11320; AAPI11735; AAPI15653 | Acetamidase | *Homo sapiens* |
| C869.01 | AAPI15236 | Amidase | *Aspergillu oryzae Emericella nidulans* |
| C869.04 | AAPI16338 | Formamidase | *Saccharomyces pombe* |
| *Aaa1* | AAPI12618; AAPI12667 | Transamidase GPI component | *Saccharomyces pombe* |
| Transamidase | AAPI12852 | PIG-S component | *Saccharomyces pombe* |
| Ceramidase | AAPI10602 | Natural ceramidase | *Homo sapiens* |
| *C550.07* | AAPI14088; AAPI14091 | Amidase | *Drosophila pseudoobscura* |
| *Nta1* | AAPI11607 | N-terminal amidase | *Saccharomyces pombe* |
| *Muramidase-2* | AAPI12478 | Muramidase-2 | *Saccharomyces pombe* |
| *Ypc1* | AAPI14069 | Allkaline ceramidase | *Enterococcus hirae* |
| *AmdR* | AAPI15935 | Acetamidase regularoty protein | *Saccharomyces cerevisiae* |
| *SPBC365.20c* | AAPI13378 | Nicotinamidase | *Aspergillu fumigatus* |
| *GPI16* | AAPI10794 | Transamidase | *Saccharomyces pombe* |
| **Esterases** | | | |
| *Otu1* | AAPI12232 | Ubiquitin thioesterase | *Saccharomyces pombe* |
| *Ppe-1* | AAPI12945 | Methylesterase-1 | *Saccharomyces* *pombe* |
| Phosphodiesterase | AAPI10444; AAPI11928 | 5-bisphosphate phosphodiesterase 1 | *Aspergillus. oryzae* |
| *Iah1* | AAPI30145 | Isoamyl acetate-hydrolyzing esterase | *Candida albicans Saccharomyces pombe* |
| Acetylesterase | AAPI11175 | Acetylesterase | *Bos taurus* |
| *Ache* | AAPI10403 | Acetilholinesterase | *Bacillus subtilis* |
| *Yeh2* | AAPI11310 | Sterol esterase 2 | *Homo sapiens* |
| *Pon2* | AAPI10054; AAPI15862 | Arylesterase 2 | *Saccharomyces cerevisiae* |
| *Pme7* | AAPI15006 | Pectinesterase inhibitor 7 | *Rattus norvegicus Gallus gallus* |
| *Pme1* | AAPI15005 | Pectinesterase 1 | *Arabidopsis thaliana* |
| *Gde1* | AAPI10289 | Phosphodiesterase | *Aspergillu tubingensis* |
| *Mpped1* | AAPI15945 | Metallophosphoesterase domain containing protein 1 | *Ashbya gosssypii* |
| *Plc* | AAPI10109 | Phosphodiesterase | *Homo sapiens* |
| *Acot8* | AAPI16350 | Acyl-coenzyme A thioesterase 8 | *Staphylococcus aureus* |
| SPAC8E11.04c | AAPI11957 | Acyl-protein thioesterase 1 | *Rattus norvegicus* |
| *Rha1* | AAPI15992; AAPI16466 | Acetylesterase | *Saccharomyces pombe* |
| *Ppt1* | AAPI16221 | Thioesterase | *Aspergillus aculeatus* |
| *Tdp 1* | AAPI10772 | Phosphodiesterase 1 | *Bos taurus* |
| SPBC725.05c | AAPI12070 | Phosphodiesterase | *Mus musculus* |
| *FaeC* | AAPI15983 | Feruloyl esterase C | *Saccharomyces pombe* |
| *YfkN* | AAPI15652 | Phosphoesterase | *Neosartorya fischeri* |
| *FaeB-2* | AAPI16330 | Esterase B-2 | *Bacillus subtilis* |
| SPCP31B10.05 | AAPI14131 | Tyrosyl-DNA phosphodiesterase | *Aspergillus flavus* |
| *Otub1* | AAPI11843 | Thioesterase | *Saccharomyces pombe* |
| *Pde-6* | AAPI11375 | Phosphodiesterase | *Homo sapiens* |
| Phosphodiesterase | AAPI14749 | 5-bisphosphate phosphodiesterase delta-4 | *Homo sapiens* |
| SPBC31F10.02 | AAPI11214; AAPI14204 | Esterase | *Macaca fascicuularis* |
| AFUA_6G02780 | AAPI14343 | Acyl-protein thioesterase 1 | *Saccharomyces pombe* |
| CNF02430 | AAPI11232 | Acyl-protein thioesterase 1 | *Aspergillu* fumigatus |
| YMR210W | AAPI16406 | Esterase | *Cryptococcus neooformans* |
| ***Proteases*** | | | |
| *Ste24* | AAPI12612 | Zinc metalloprotease CAAX prenyl protease, family M48B | *Saccharomyces cerevisiae* |
| SPAC1687.02 | AAPI12796 | Endoprotease CAAX prenyl protease 2 | *Saccharomyces pombe* |
| Zinc metalloprotease | AAPI12953 | Metallopeptidase family M28 family | *Coccidioides immitis* |
| Zinc metalloprotease | AAPI13100 | Zinc metalloprotease | *Coccidioides immitis* |
| *Mug138* | AAPI12729; AAPI13964 | Zinc metalloprotease | *Saccharomyces cerevisiae* |
| *Senp3* | AAPI30104 | Sentrin-specific protease | *Saccharomyces pombe* |
| *Ulp2* | AAPI11402 | Ubiqutin-like specific protease 2 | *Mus musculus* |
| *Ulp1* | AAPI16547; AAPI30014 | Ubiqutin-like specific protease 1 | *Saccharomyces pombe* |
| *Pfpl* | AAPI30044 | Intracellular protease | *Saccharomyces pombe* |
| *Sst2* | AAPI13597 | AMSH-like protease | *Pyrococcus abyssi* |
| F5610.1 | AAPI14398 | Serine protease | *Caenorhabditis elegans* |
| *Nma11* | AAPI30019; AAPI10369 | Pro-apoptotic serine protease | *Ajellomyces capsulatus* |
| K12H4.7 | AAPI13861; AAPI14349 | Serine protease | *Caenorhabditis elegans* |
| *PalB* | AAPI11731; AAPI11730 | Calpain-like protease | *Aspergillus oryzae* |
| *Cym1* | AAPI16168 | Mitochondrial presequence protease, | *Aspergillus oryzae* |
| *Imp2* | AAPI11686 | Mitochondrial inner membrane protease subunit 2 | *Aspergillus fumigatus* |
| *Imp1* | AAPI14795 | Mitochondrial inner membrane protease subunit 1 | *Saccharomyces cerevisiae* |
| *Atp23* | AAPI12854 | Mitochondrial inner membrane protease | *Saccharomyces cerevisiae* |
| *Oma1* | AAPI16028; AAPI11644 | Mitochondrial metallopeptidase | *Coccidioides immitis* |
| *Dug1* | AAPI14375 | Cys-Gly metallopeptidase | *Saccharomyces pombe* |
| *Pim1* | AAPI11741 | Lon protease, | *Neurospora crassa* |
| *ClpP* | AAPI14293 | ATP-dependant Clp protease proteolytic subunit | *Anaplasma marginale* |
| *ClpX* | AAPI14801 | ATP-dependant Clp protease ATP-binding subunit | *Methylococcus capsulatus* |
| *Clp1* | AAPI15379 | ATP-dependant Clp protease proteolytic subunit 1 | *Methylococcus capsulatus* |
| *Pep2* | AAPI30157; AAPI14240 | Secreted endopeptidase pep2 | *Aspergillus fumigatus* |
| *Kex1* | AAPI11807 | Carboxypeptidase | Contributes to virulence *Aspergillus fumigatus* |
| *Cpy1* | AAPI15998 | Carboxypeptidase | *Candida albicans* |
| *Oct1* | AAPI12984 | Mitocondrial peptidase | *Candida albicans* |
| *Ggt1* | AAPI10489 | Gamma-glutamyltranspeptidase | *Saccharomyces pombe* |
| *Sed1* | AAPI10528; AAPI12045 | Tripeptidyl-peptidase | *Aspergillus fumigatus* |
| *Ape1* | AAPI15352; AAPI16234 | Vacuolar aminopeptidase 1 | *Aspergillus fumigatus* |
| *Kae1* | AAPI16411 | Glycoprotein endopeptidase | *Aspergillus fumigatus* |
| *Lap1* | AAPI10100 | Leucine aminopeptidase 1 | *Trichophyton rubrum* |
| C25B8.17 | AAPI14880 | Intramembrane protease | *Aspergillus fumigatus* |
| AFUA_5G111750 | AAPI10306 | Lon protease homolog 2 |  |
| *Atg4* | AAPI12506 | Cysteine protease | *Coccidioides immitis* |
| C2E11.08 | AAPI15707 | Dipeptidyl aminopeptidase | *Saccharomyces pombe* |
| C965.12 | AAPI16162 | Dipeptidase | *Saccharomyces pombe* |
| SPBC3E7.10 | AAPI14780 | Methionine aminopeptidase 1 | *Saccharomyces pombe* |
| *Amp1* | AAPI13776; AAPI13778 | Glutamate carboxypeptidase 2 | *Arabidopsis thaliana* |
| Peptidase | AAPI16376 | LON peptidase, N-terminal domain |  |
| **Lipases** | | | |
| *Plc3* | AAPI14097 | Phospholipase C3 | *Micobacterium tuberculosis* |
| AFUA_1G04970 | AAPI15828 | Patatin-like phospholipase domain-containing protein | *Micobacterium tuberculosis* |
| CIMG_04897 | AAPI14261 | Patatin-like phospholipase domain-containing protein | *Coccidioides immitis* |
| NFIA_019760 | AAPI13746 | Patatin-like phospholipase domain-containing protein | *Neosartorya ficheri* |
| *Pld* | AAPI16121 | Phospholipase D | *Micobacterium tuberculosis* |
| *Pld2* | AAPI12191 | Phospholipase D2 | *Phytophtora infestans* |
| *Pld1* | AAPI11727 | Phospholipase D1 | *Phytophtora infestans* |
| *Pla2g4d* | AAPI10455 | Cytosolic phospholipase A2 | *Phytophtora infestans* |
| YOR022C | AAPI14210 | Phospholipase | *Saccharomyces cerevisiae* |
| PlcN | AAPI14561 | Non-hemolyic phospholipase C | *Saccharomyces cerevisiae* |
| SPCC 1020.13c | AAPI11593 | Phospholipase | *Saccharomyces cerevisiae* |
| C16A3.12c | AAPI11669 | Lipase | *Saccharomyces cerevisiae* |
| *Lipe* | AAPI11318 | Hormone sensitive lipase | *Bos taurus* |
| *Tgl2* | AAPI10592 | Lipase 2 | *Saccharomyces cerevisiae* |
| *Lip3* | AAPI11984 | Lipase 3 | *Saccharomyces cerevisiae* |
| YOR059C | AAPI13164 | Lipase | *Penicillium chrysogenum* |
| *Atg 15* | AAPI10862 | Lipase | *Coccidioides immitis* |
| *Nte1* | AAPI12225; AAPI12244 | Lysophospholipase | *Penicillium chrysogenum* |
| Lysophospholipase | AAPI16254 | Lysophospholipase | *Rattus norvegicus* |
| *Aspg* | AAPI16575 | 60 kDa Lysophospholipase | *Rattus norvegicus* |
| *Lip2* | AAPI14588 | Lipase 2 | *Saccharomyces cerevisiae* |
| *Lip3* | AAPI30183 | Lipase 3 | *Yarrowia lipolytica* |
| *Lip4* | AAPI13536  AAPI10881  AAPI13544 | Lipase 4 | *Candida rugosa* |
| *Rog1* | AAPI10845 | Lipase | *Saccharomyces cerevisiae* |
